# Species Identification of *Conyza bonariensis* Assisted by Chloroplast Genome Sequencing

**DOI:** 10.3389/fgene.2018.00374

**Published:** 2018-09-11

**Authors:** Aisuo Wang, Hanwen Wu, Xiaocheng Zhu, Jianmin Lin

**Affiliations:** ^1^Wagga Wagga Agricultural Institute, NSW Department of Primary Industries, Wagga Wagga, NSW, Australia; ^2^Graham Centre for Agricultural Innovation (An alliance between NSW Department of Primary Industries and Charles Sturt University), Wagga Wagga, NSW, Australia; ^3^College of Mathematical Sciences, Huaqiao University, Quanzhou, China

**Keywords:** *Conyza bonariensis*, chloroplast genome, DNA barcoding, NGS, Illumina

## Abstract

Flaxleaf fleabane (*Conyza bonariensis* [L.] Cronquist) is one of the most difficult weeds to control worldwide. There are more than 150 *Conyza* species in the world and eight species in Australia. Correct identification of these species can be problematic due to their morphological similarities especially at seedling stage. Developing a robust genetics – based species identification method to distinguish *C. bonariensis* from other closely related species is important for early control of weeds. We thus examined the chloroplast (cp) genome of *C. bonariensis*, aiming to identify novel DNA barcodes from the genome sequences, and use the entire cp genome as a super-barcode for molecular identification. The *C. bonariensis* chloroplast genome is 152,076 bp in size, encodes 133 genes including 88 protein-coding genes, 37 tRNA genes and 8 ribosomal RNA genes. A total of 151 intergenic regions and 19 simple sequence repeats were identified in the cp genome of *C. bonariensis*, which provides a useful genetic resource to develop robust markers for the genetic diversity studies of *Conyza* species. The sequence information was used to design a robust DNA barcode *rps*16 and *trnQ-UUG* which successfully separated three predominant *Conyza* species (*C. bonariensis*, *C. canadensis*, and *C. sumatrensis*). Phylogenetic analyses based on the cp genomes of *C. bonariensis*, *C. canadensis* and 18 other Asteraceae species revealed the potential of using entire cp genome as a plant super-barcode to distinguish closely-related weed species.

## Introduction

Flaxleaf fleabane or hairy fleabane (*Conyza bonariensis* [L.] Cronquist) (synonym: *Erigeron* *bonariensis* L.) is native to South America. It was first described from Argentina (Michael, 1977) and naturalizes in warm areas throughout the world (Wu, 2009). Broadly, *Conyza* Less. belongs to the daisy family Asteraceae, tribe Astereae, and subtribe Conyzinae (Funk et al., 2009). Both *Conyza* and *Erigeron* are classified in Conyzinae (Nesom, 2008). Molecular studies showed that *Conyza* and several other genera are derived from within the genus *Erigeron* (Noyes, 2000) although the generic classification is still debated (Brouillet et al., 2009). Morphologically *Conyza* differs from *Erigeron* in reduced ligule length of ray florets and decreased number of hermaphroditic disk florets relative to female ray florets, while most species of *Erigeron* have conspicuous ray and relatively numerous disk florets (Noyes, 2000). In Anglo-American countries, *Conyza* is mostly treated as a separate, polyphyletic genus following Cronquist (1943) and Noyes (2000). In Europe, *Conyza* is now lumped with *Erigeron* (Greuter, 2003).

There are more than 150 *Conyza* species worldwide (Flann, 2016) and 8 species in Australia (Alpen, 2014). *Conyza* spp. are highly invasive weeds in more than 40 different crops in 70 countries (Holm et al., 1997; Hao et al., 2009). Among those weeds, *C. bonariensis, C. canadensis*, and *C. sumatrensis* are the most widespread species globally (Thebaud and Abbott, 1995; Weaver, 2001) and have evolved resistance to glyphosate in many countries (Heap, 2018).

*Conyza bonariensis* is an annual or short-lived perennial plant. In Australia, it was first reported as a weed in the 1980s, and has since become one of the most difficult weeds to control. It is rated 4 on a scale of 0–5 as a weed affecting natural ecosystems and a highest rating of 5 in agricultural ecosystems (Groves et al., 2003). *Conyza bonariensis* can occur in most soil types and plant communities, particularly in areas of disturbed soil and in and around gardens (Cunningham et al., 1981). Wu et al. (2010) reported that *C. bonariensis* reduces sorghum yield by 65 to 98% if not controlled.

The invasiveness of *C. bonariensis* is associated with its high fecundity, high potential level of dispensability, staggered emergence, and resistance to the herbicide glyphosate (Wu et al., 2007). The increasing abundance of *C. bonariensis* is associated with the adoption of no-till farming systems, partly due to the lack of cultivation and a better environment for germination and seedling survival as a result of stubble retention. The achene (i.e., a one-seeded indehiscent fruit; hereinafter for simplicity referred to as seed) is very sensitive to burial, with most seedling emergence occurring from the soil surface or from a burial depth of 0.5 cm (Wu et al., 2007). It is very prolific, producing 119,000–266,800 seeds per plant (Kempen and Graf, 1981; Wu et al., 2007). Among the mature seeds produced, 80% are viable and non-dormant, capable of germination soon after shedding. Another characteristic contributing to its invasiveness is the low settling velocity of the small, light-weighted seed equipped with a pappus (Andersen, 1992), indicating the seed may travel some distance before settling onto the ground. These key biological features indicate that the spread of *C. bonariensis* across agricultural landscapes could be very rapid and facilitated by long distance dissemination via wind, surface runoff, and water movement in irrigation channels and waterways.

Taxonomic confusions between *C. bonariensis C. canadensis, C. sumatrensis*, and other *Conyza* species are very common (Michael, 1977; Wu, 2009; Marochio et al., 2017), especially at young seedling stage. Although taxonomic keys are available for the identification of *Conyza* species in Australia (Michael, 1977; Everett, 1992), it has been a challenging task to distinguish these species due to overlapping morphological features. The correct identification requires taxonomic expertise and relies heavily on the availability of flowering materials and other morphological characters. DNA barcoding was thus applied to identify *C. bonariensis* from other *Conyza* species at early growth stage based on eight DNA barcodes selected from limited gene sequences in GenBank (Alpen et al., 2014). While this approach confirmed the usefulness of a two-locus DNA barcode (ITS *– rbcL*) for potential identification of *Conyza* species at the species level, it failed to distinguish *C. bonariensis* from *C. bilbaoana*, which highlighted the necessity of seeking more robust approaches for better identification of *C. bonariensis* from other *Conyza* species at early growth stage.

Correct species identification is a fundamental step in developing effective strategies for *Conyza* control. Misidentification could result in poor control of the target species (Marochio et al., 2017). The genetic differences between weed species can also affect the choice and efficacy of biological control agents (Dekker, 1997). *Conyza* species vary in their biology, phenology and susceptibility to control options (Thebaud and Abbott, 1995; Weaver, 2001; Wu, 2009; González-Torralva et al., 2010). For example, the sensitivities to glyphosate were ranked in decreasing order as *C. sumatrensis*, *C. bonariensis*, and *C. canadensis* (González-Torralva et al., 2010). Moretti and Hanson (2016) also reported that the absorption of glyphosate is higher in *C. bonariensis* than in *C. canadensis*. The unique biology and phenology of each *Conyza* species can be used to determine the weakest link, resulting in better controlling timing and more efficient control.

Using entire chloroplast (cp) genome as a plant super-barcode to distinguish closely related species has been reported (Parks et al., 2009; Kane et al., 2012; Dodsworth, 2015). Compared to the traditional DNA barcode approaches using single or two loci, a cp genome super-barcode has multiple advantages. It has more informative sites for species discrimination. It can separate two species by detecting gene loss or defining gene order, which is impossible in traditional DNA barcoding (Luo et al., 2008, 2009). Furthermore, the analysis of this super-barcode can avoid problems encountered by traditional barcoding studies such as the lack of universal primers, the low PCR success rate and the amplification of pseudogenes (Song et al., 2008). In addition, the availability of cp genome can reveal novel loci and intergenic regions that can improve the species delineation by the traditional DNA barcoding approach.

Up to now (May 2, 2018), a total of 82 complete cp genomes have been deposited into GenBank for the tribe of Astereae. Among these cp genomes, only one was from the genus *Conyza* (*Conyza bonariensis* Q17-R9) (Hereward et al., 2017). While a draft nuclear genome of *Conyza canadensis* (synonym: *Erigeron canadensis*) has been reported (Peng et al., 2014), the whole cp genome sequence of this species is not publically accessible at GenBank, but can be found in the **Supplementary Data** of the paper (Peng et al., 2014).

Here, we sequenced the cp genome of a *C. bonariensis* specimen (Wagga Wagga, NSW, Australia) using the Illumina Hiseq 2000 platform, and incorporated the sequences in a dataset covering all complete chloroplast sequences from the tribe Astereae. One representative per genus was selected for further phylogenetic analysis. The generated cp genome sequences are expected to provide helpful genetic resources to conduct DNA barcoding and molecular phylogenetic studies of *C. bonariensis*, thereby contributing to genetic and evolutionary studies and the subsequent management of *Conyza* weeds.

## Materials and Methods

### Sample Collection, Genomic DNA, Extraction, and Sequencing

A wild *C. bonariensis* sample was collected from Wagga Wagga, NSW, Australia (35°7′8″ S and 147°22′8″ E). The voucher sample has been deposited into Wagga Wagga Agricultural Institute (WWAI) (Voucher No. WW08606). Fresh leaves of *C. bonariensis* WW08606 were cleaned, frozen in liquid nitrogen, and ground to fine powder prior to the DNA extraction. A modified CTAB protocol (Doyle and Doyle, 1987) was applied to extract total genomic DNA from the leaf tissues. Briefly, the leaf tissue was digested in CTAB extraction buffer [100 mM Tris-HCl (pH 7.5), 25 mM EDTA, 1.5 M NaCl, 2% (w/v) CTAB] at 55°C overnight before being extracted twice with chloroform: isoamyl alcohol (24:1). About 10% volume of 5 M NaCl was added into the supernatant before being precipitated with 100% ethanol. DNAs that met the QC requirements were used for the construction of paired-end library according to the standard protocol (Illumina, San Diego, CA, United States). Sequence analyses were carried out with a HiSeq 2000 sequencer (Illumina) in the PE sequencing mode (125-bases each) at Beijing Genomics Institute (BGI, Hong Kong).

### Chloroplast Genome Assembly and Annotation

The paired end run produced 13,324,346 reads for each direction, which gave more than 100-fold coverage of the chloroplast genome. The super-fast FASTA/Q file manipulation tool, readfq v5^1^, was applied to trim low quality reads, adapter sequences and duplicate sequences in the raw reads of Illumina sequencing data. The quality – filtered reads were subject to *de novo* assembling with SOAPdenovo2 (the k-mer size was optimized to 61) (Luo et al., 2012). The resultant scaffolds were subjected to gap-filling with the Illumina reads by using GapCloser 1.10 (*P* = 31^2^). Annotation of the *C. bonariensis* chloroplast genome was performed using CpGAVAS with default settings (Liu et al., 2012). The predicted annotations were verified using BLAST similarity search (Altschul et al., 1990). In addition, all tRNA genes were further verified using tRNAscan-SE search server (Lowe and Eddy, 1997)^3^. All annotations were manually edited for incorrect stop and start locations before submission to GenBank (Accession No. KX792499). The circular *C. bonariensis* chloroplast genome map was drawn using OGDraw v1.2 (Lohse et al., 2007).

### Comparative Genome Analysis

The whole cp genome of *C. bonariensis* WW08606 was compared to the published cp genome of *C. bonariensis* Q17-R9 (MF276802), and the cp genome of *C. canadensis* (Peng et al., 2014). GMATo (Wang et al., 2013) was applied to search simple sequence repeats (SSRs) in the genomes with the default parameters (-r 5 -m 2 -x 10 -s 0 -i). The three cp genomes were aligned using progressive MAUVE (Darling et al., 2004) at default settings for gene arrangement comparison. The whole cp genome sequences of three *Conyza* species and other 18 Astereae species, including *Archibaccharis asperifolia* (KX063859.1), *Aztecaster matudae* (KX063935.1), *Baccharis tricuneata* (KX063888.1), *Blakiella bartsiifolia* (KX063886.1), *Diplostephium alveolatum* (KX063856.1), *Exostigma notobellidiastrum* (KX063881.1), *Floscaldasia hypsophila* (KX063916.1), *Helianthus annuus* (CM007907.1), *Heterothalamus alienus* (KX063869.1), *Hinterhubera ericoides* (KX063910.1), *Laennecia sophiifolia* (KX063899.1), *Laestadia muscicola* (KX063873.1), *Lagenophora cuchumatanica* (KX063879.1), *Llerasia caucana* (KX063908.1), *Oritrophium peruvianum* (KX063861.1), *Parastrephia quadrangularis* (KX063923.1), *Pityopsis falcata* (KY045817.1), and *Westoniella kohkemperi* (KX063921.1), were aligned in MAFFT at default settings (Katoh et al., 2005). The resulting alignment file was applied to construct a maximum likelihood (ML) tree under the GTR nucleotide substitution model (plus Gamma distribution) in PhyML 3.1 (Guindon et al., 2009). Clade support was assessed via non-parametric bootstrapping using 1000 bootstrap replicates.

### DNA Barcoding of *Conyza* Species With Primers Designed From the cp Genome of *C. bonariensis*

A pair of primers (forward: AGACATTACTTCGGTGCT; reverse: TAGAAAGCAACGTGCGACTT) was designed based on the intergenic region *of rps16* and *trnQ-UUG* in the cp genome of *C. bonariensis* (ww08606), and synthesized by Sigma-Aldrich in Australia. The primer was applied to sequence a total of 13 *Conyza* specimens, including four *C. bonariensis*, four *C. canadensis*, four *C. sumatrensis*, and one *C. bilbaoana* (**Supplementary Table S1**). The resulting DNA sequences were subject to construct a ML tree using the GTR model in MEGA 7 (Kumar et al., 2016). Clade support was again assessed via non-parametric bootstrapping with 1000 bootstrap replicates.

## Results and Discussion

### General Features of the *C. bonariensis* WW08606 Chloroplast Genome

**FIGURE 1:**
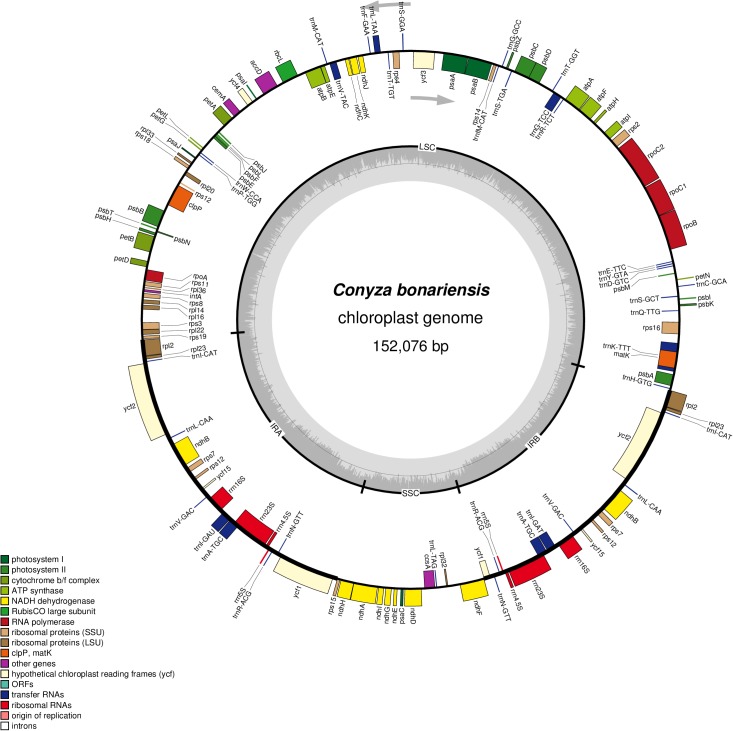
Sequence map of the cp genome of *Conyza bonariensis* WW08606. Genes drawn outside the circle are transcribed clockwise, while genes shown inside the circle are transcribed counter-clockwise. Genes belonging to different functional groups are color-coded. The darker gray in the inner circle indicates GC content, while the lighter gray corresponds to AT content. IRa, inverted repeat A; IRb, inverted repeat B; LSC, large single copy; SSC, small single copy.

The obtained *C. bonariensis* WW08606 cp genome was 152,076 bp in size, encoded 133 genes including 88 protein-coding genes, 37 tRNA genes, and 8 ribosomal RNA genes. The total GC content of the genome was 37.16%, which is in agreement with the cp genome of *C. bonariensis* Q17-R9 and *C. canadensis* (**Table 1**). AT-rich areas in the *C. bonariensis* WW08606 cp genome were the intergenic regions (67.57%) and protein-coding genes (62.13%), while a much lower AT content was found in rRNAs (45.88%) and tRNAs (47.65%) genes. This pattern is also similar to that of *C. bonariensis* Q17-R9 and *C. canadensis* (**Table 1**). The annotated cp genome of *C. bonariensis* WW08606 is shown in **Figure 1**. The whole genome was divided into four regions, including one long single copy section (LSC), one short single copy section (SSC) and two inverted repeat (IR) sections (IRa and IRb). All rRNA genes were located in the IR regions, whilst tRNA and protein coding genes were present across all sections.

**Table 1:** The cp genome features of *C. bonariensis* WW08606, *C. bonariensis* Q17-R9, and *C. canadensis.*

Characteristics	*C. bonariensis* WW08606	*C. bonariensis* Q17-R9	*C. canadensis*
GenBank Accession No.	KX792499	MF276802	Nil
Size (bp)	152,076	153,014	152,639
GC content (%)	37.16	37.16	37.14
Total number of genes	133	132	142
Protein-coding genes	88	87	95
Ribosomal RNAs	8	8	8
Transfer RNAs	37	37	39
**AT content (%)**			
Genome	62.84	62.84	62.86
Protein-coding genes	62.13	62.18	62.34
Ribosomal RNAs	45.88	44.83	45.89
Transfer RNAs	47.65	46.33	49.34
Intergenic region (bp)	67.57	67.62	67.81

### Genome Structure Comparison Between *C. bonariensis* WW08606, *C. bonariensis* Q17-R9, and *C. canadensis*

**FIGURE 2:**
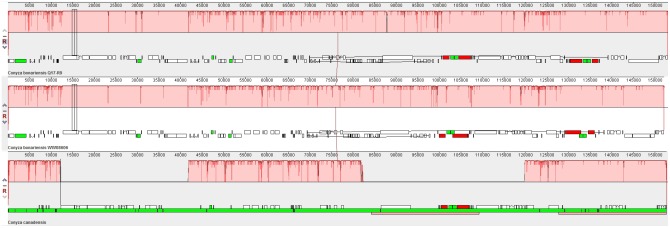
Alignment of three *Conyza* cp genomes in MAUVE. The identified locally collinear blocks (LCBs) for each genome are labeled with pink color. The gene order of each cp genome is shown below the LCBs.

Our MAUVE analyses revealed that the cp genome of *C. bonariensis* WW08606, while being different from the cp genome of *C. canadensis* in terms of the number of locally collinear blocks (LCBs), was similar to that of *C. bonariensis* Q17-R9. The two *C. bonariensis* cp genomes had the same number of LCBs and the same direction of transcriptions (**Figure 2**).

As each LCB represents a conserved segment that appears to be internally free from genome rearrangements (Darling et al., 2004), the different number of LCBs between *C. bonariensis* and *C. canadensis* could suggest a difference in gene order between two species. Similarly, the nearly identical LCBs structure between the two strains of *C. bonariensis* indicates the conservation of gene order within the same species. This finding thus supports the use of whole cp genomes as a super barcode for species discrimination as it reveals not only interspecific differences but also intraspecific similarities from the angle of gene order.

By examining the gene content of the two *C. bonariensis* cp genomes, we found two *ycf15* genes in *C. bonariensis* WW08606 and one *ycf15* gene in *C. bonariensis* Q17-R9. The presence of two *ycf15* genes is common in other Astereae cp genomes (blast results). Further blast analysis revealed the presence of the second *ycf15* gene in the cp genome of *C. bonariensis* Q17-R9 (located in the region between positions 137786 and 137977), suggesting that the single *ycf15* gene structure in the published cp genome of *C. bonariensis* Q17-R9 could be an error of annotation.

### Intergenic Regions in *C. bonariensis* WW08606

*Conyza bonariensis* WW08606 contained 151 intergenic regions, in which 10 intergenic regions were greater than 1,000 bp in size, whilst 29 other intergenic regions were between 500 and 1,000 bp in size (**Supplementary Table S2**). This made it possible to screen promising DNA barcodes for the differentiation of *C. bonariensis* WW08606 from other *Conyza* species because intergenic regions have been widely used as plastid barcodes for species differentiation (Dong et al., 2012; Suzuki et al., 2014), particularly for those with highly variable sequence regions and in favorable size between 500 and 1,000 bp.

**FIGURE 3:**
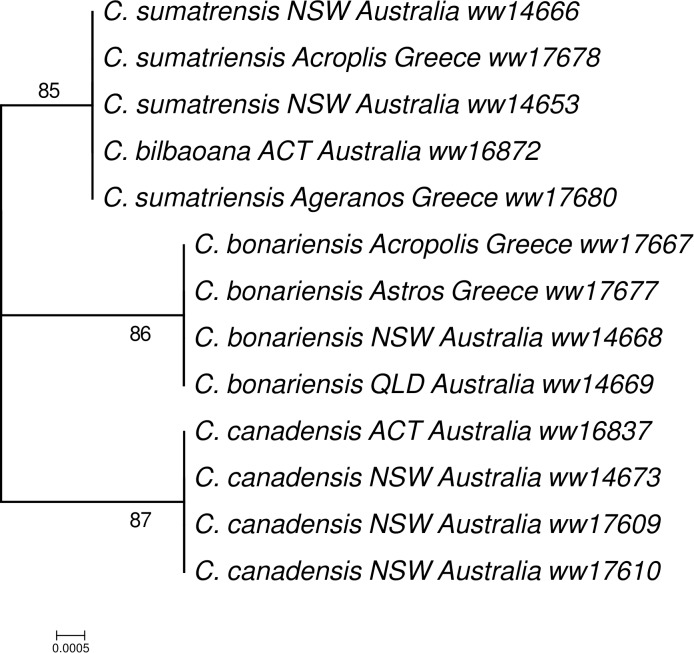
Maximum likelihood (ML) phylogenetic tree inferred from the *rps16* – *trnQ-UUG* intergenic spacer across 13 samples of *Conyza* species.

Based on the intergenic regions of *C. bonariensis*, we have designed several primers for DNA barcoding of *Conyz*a species. Among these primers, the primer designed from the intergenic region of *rps16* and *trnQ-UUG* yielded promising results. The ML tree inferred from this region across 13 *Conyza* specimens, collected from Australia and Greece, clustered all the *C. bonariensis* specimens into a monophyletic clade with high bootstrap value (86, **Figure 3**). This clade was clearly separated from the other two clades: the clade of *C. canadensis* and the clade of *C. sumatrensis/C. bilbaoana*, highlighting the DNA barcode potentials of this maker for *Conyza* species delineation. It successfully separated *C. bonariensis* and *C. bilbaoana*, which has been a problem in a previous DNA barcoding study (Alpen et al., 2014). Our results provided more evidence for using *rps16-trnQ* in barcoding and phylogenetics studies for Asteraceae species (Laureto and Barkman, 2011; Salih et al., 2017).

### Simple Sequences Repeats in *C. bonariensis* WW08606 cp Genome

Simple sequence repeats in *C. bonariensis* WW08606 were detected using GMATo (Wang et al., 2013). Dinucleotide repeats were found to be the most common type of SSR in the cp genome (**Table 2**). The most frequent SSR motif in *C. bonariensis* was ‘TA,’ which was found in nine locations with repetition rate at five, six, or seven. This is followed by the SSR motif of ‘AT,’ which was repeated at five, six or seven times in eight locations across the genome. SSR loci proved to be valuable in studying the genetic diversity of plants, including weeds (El-Esawi et al., 2016; Feng et al., 2016). Further study is thus required to screen promising DNA markers from these SSR sites.

**Table 2:** SSR (simple sequence repeats) statistics of *C. bonariensis* WW08606.

Start position	End position	Repetitions	Motif
109636	109650	5	GAA
18722	18731	5	TA
186	195	5	TA
19720	19729	5	AT
30949	30958	5	TA
35305	35314	5	AT
36001	36014	7	AT
4829	4843	5	TTA
52373	52384	6	AT
59279	59288	5	TA
59333	59344	6	AT
59376	59385	5	AT
59398	59409	6	TA
60753	60762	5	TA
61257	61270	7	AT
62286	62299	7	TA
68053	68062	5	TA
77615	77624	5	TA
82308	82317	5	AT

### Phylogenetic Analysis of *C. bonariensis*, *C. canadensis*, and Other Species From the Astereae Tribe

**FIGURE 4:**
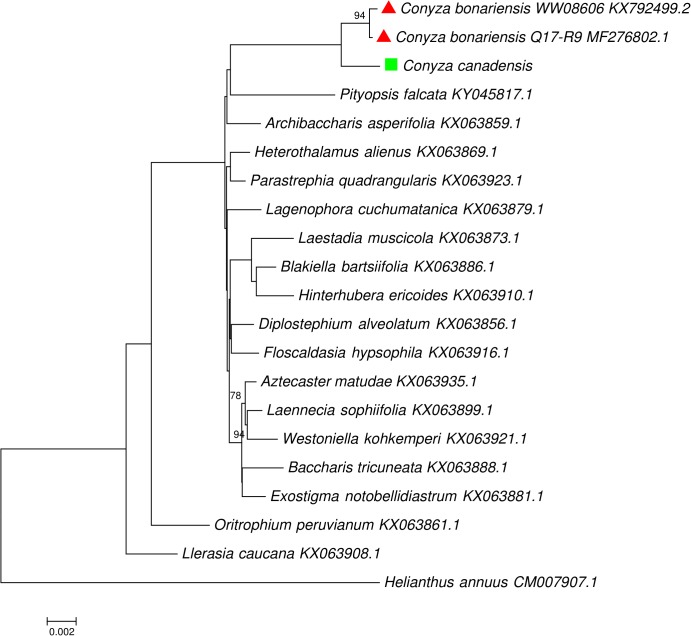
Maximum likelihood phylogenetic tree inferred from complete chloroplast genomes from tribe Astereae, one representative per genus (three cp genomes for *Conyza*), with *Helianthus annuus* as the outgroup. Taxon names of *C. bonariensis* and *C. canadensis* were labeled with red triangle and green square, respectively.

The availability of cp genome of *C. bonariensis* made it possible to explore the phylogenetic relationship of this weed against other Astereae species at the whole cp genome scale. Our phylogenetic analyses supported the results of Hereward et al. (2017) by confirming the monophyletic status of the group of *Exostigma notobellidiastrum*, *Baccharis tricuneata*, *Aztecaster matudae*, *Laennecia sophiifolia*, and *Westoniella kohkemperi*; the group of *Laestadia muscicola*, *Blakiella bartsiifolia*, and *Hinterhubera ericoides*; the group of *Heterothalamus alienus* and *Parastrephia quadrangularis*, and the group of *Diplostephium alveolatum* and *Floscaldasia hypsophila* (**Figure 4**).

In our phylogenetic analysis, three *Conyza* specimens were separated from other Astereae species by forming a monophyletic clade (**Figure 4**). Within this clade, two *C. bonariensis* specimens formed a sub-clade with strong bootstrap value support (94). This topology shows the separation of *Conyza* at both the inter- and intra- species level, indicating the potentials of using whole cp genome as a super-barcode for the separation of closely related *Conyza* species. With more cp genomes of *Conyza* species being sequenced in the future, we believe that the super-barcode approach using the whole cp genome will become increasingly valuable in species identification.

## Conclusion

The present study examined the cp genome of *C. bonariensis*. The cp genome was 152,076 bp in size, encoded 133 genes including 88 protein-coding genes, 37 tRNA genes and 8 ribosomal RNA genes. A total of 151 intergenic regions and 19 SSR were identified in the cp genome of *C. bonariensis*. These results are in agreement with the previous study by Hereward et al. (2017). The sequence information has enabled us to design a robust DNA barcode *rps16* and *trnQ-UUG* which successfully separated *C. bonariensis* from *C. canadensis* and *C. sumatrensis/C. bilbaoana*. The abundant genetic sequence information provides a rich genetic resource for further development of robust markers for genetic diversity and evolutionary studies of *Conyza* species. The phylogenetic studies based on the whole cp genomes of *C. bonariensis*, *C. canadensis* and 18 other Astereae species revealed the potential of using the entire cp genome as a plant super-barcode to distinguish closely-related weed species.

## Author Contributions

HW designed the research. AW conducted the research and drafted the manuscript. AW and JL analyzed the data. XZ conducted the DNA barcoding work. All authors reviewed and approved the manuscript.

## Conflict of Interest Statement

The authors declare that the research was conducted in the absence of any commercial or financial relationships that could be construed as a potential conflict of interest.The reviewer CP and handling Editor declared their shared affiliation.

## Footnotes

1 https://github.com/lh3/readfq

2 http://soap.genomics.org.cn

3 http://lowelab.ucsc.edu/tRNAscan-SE

## References

[B1] Alpen K. (2014). *The Development of a DNA Barcode System for Species Identification of Conyza* Spp. Wagga Wagga, NSW: Charles Sturt University.

[B2] Alpen K. Gopurenko D. Wu H. Lepschi B. J. Weston L. A. (2014). “The development of a DNA barcode system for species identification of *Conyza* spp. (fleabane),” in *Proceedings of the 19th Australasian Weeds Conference – Science, Community and Food Security: the Weed Challenge*, Hobart, 401–404.

[B3] Altschul S. F. Gish W. Miller W. Myers E. W. Lipman D. J. (1990). Basic local alignment search tool. *J. Mol. Biol.* 215 403–410. 10.1016/S0022-2836(05)80360-2 2231712

[B4] Andersen M. (1992). An analysis of variability in seed settling velocities of several wind- dispersed Asteraceae. *Am. J. Bot.* 79 1087–1091. 10.1002/j.1537-2197.1992.tb13702.x 30139130

[B5] Brouillet L. Lowrey T. Urbatsch L. Karaman-Castro V. Sancho G. Wagstaff S. (2009). “Phylogeny, classification, and biogeography of the Astereae,” in *Systematics, Evolution and Biogeography of the Compositae*, eds Funk V. A. Susanna A. Stuessy T. Bayer R. (Vienna: IAPT), 589–629.

[B6] Cronquist A. (1943). The separation of erigeron from conyza. *Bull. Torrey Bot. Club* 70 629–632. 10.2307/2481719

[B7] Cunningham G. H. Mulham W. E. Milthorpe P. L. Leigh J. H. (1981). *Plants of Western New South Wales.* Sydney, NSW: NSW Government Printing Office, Soil Conservation Service of NSW, 662.

[B8] Darling A. C. Mau B. Blattner F. R. Perna N. T. (2004). Mauve: multiple alignment of conserved genomic sequence with rearrangements. *Genome Res.* 14 1394–1403. 10.1101/gr.2289704 15231754 PMC442156

[B9] Dekker J. (1997). Weed diversity and weed management. *Weed Sci.* 45 357–363.

[B10] Dodsworth S. (2015). Genome skimming for next-generation biodiversity analysis. *Trends Plant Sci.* 20 525–527. 10.1016/j.tplants.2015.06.012 26205170

[B11] Dong W. P. Liu J. Yu J. Wang L. Zhou S. L. (2012). Highly variable cp markers for evaluating plant phylogeny at low taxonomic levels and for DNA barcoding. *PLoS One* 7:e35071. 10.1371/journal.pone.0035071 22511980 PMC3325284

[B12] Doyle J. J. Doyle J. L. (1987). A rapid DNA isolation procedure for small quantities of fresh leaf tissue. *Phytochem. Bull.* 19 11–15.

[B13] El-Esawi M. A. Germaine K. Bourke P. Malone R. (2016). Genetic diversity and population structure of *Brassica oleracea* germplasm in Ireland using SSR markers. *C. R. Biol.* 339 133–140. 10.1016/j.crvi.2016.02.002 26995396

[B14] Everett J. (1992). “Conyza,” in *‘Flora of New South Wales’* Vol. 3 ed. Harden G. J. (Sydney, NSW: New South Wales University Press), 197–200.

[B15] Feng S. He R. Lu J. Jiang M. Shen X. Jiang Y. (2016). Development of SSR markers and assessment of genetic diversity in medicinal *Chrysanthemum morifolium* Cultivars. *Front. Genetics.* 7:113. 10.3389/fgene.2016.00113 27379163 PMC4908101

[B16] Flann C. (ed.) (2016). “GCC: global compositae checklist (version 5 (Beta), Jun 2014,” in *Species 2000 and ITIS Catalogue of Life*, eds Roskov Y. Abucay L. Orrell T. Nicolson D. Kunze T. Flann C. (Leiden: Naturalis).

[B17] Funk V. A. Susanna A. Stuessy T. F. Robinson H. (2009). “Classification of compositae,” in *Systematics, Evolution, and Biogeography of Compositae*, eds Funk V. A. Susanna A. Stuessy T. F. Bayer R. J. (Vienna: International Association for Plant Taxonomy (IAPT)), 171–192.

[B18] González-Torralva F. Cruz-Hipolito H. Bastida F. Mülleder N. Smeda R. J. De Prado R. (2010). Differential susceptibility to glyphosate among the *Conyza* weed species in Spain. *J. Agric. Food Chem.* 58 4361–4366. 10.1021/jf904227p 20225860

[B19] Greuter W. (2003). The euro+med treatment of astereae (Compositae) - generic concepts and required new names. *Willdenowia* 33 45–47. 10.3372/wi.33.33103

[B20] Groves R. H. (Convenor) Hosking J. R. Batianoff G. N. Cooke D. A. Cowie I. D. Johnson R. W. (2003). *Weed Categories for Natural and Agricultural Ecosystem Management.* Canberra, ACT: Bureau of Rural Sciences.

[B21] Guindon S. Delsuc F. Dufayard J. F. Gascuel O. (2009). Estimating maximum likelihood phylogenies with PhyML. *Methods Mol. Biol.* 537 113–137. 10.1007/978-1-59745-251-9_6 19378142

[B22] Hao J. H. Qiang S. Liu Q. Q. Cao F. (2009). Reproductive traits associated with invasiveness in *Conyza sumatrensis*. *J. Syst. Evol.* 47 245–254. 10.1111/j.1759-6831.2009.00019.x

[B23] Heap I. (2018). *The International Survey of Herbicide Resistant Weeds.* Available at: http://www.weedscience.com/ [accessed July 6, 2018].

[B24] Hereward P. J. Werth A. J. Thornby F. D. Keenan M. Chauhan S. B. Walter H. G. (2017). Complete chloroplast genome of glyphosate resistant *Conyza bonariensis* (L.) Cronquist from Australia. *Mitochondrial DNA B* 2 444–445. 10.1080/23802359.2017.1357441 PMC7799704 33473856

[B25] Holm L. Doll J. Holm E. Pancho J. Herberger J. (1997). *World Weeds, Natural Histories and Distribution.* New York, NY: John Wiley.

[B26] Kane N. Sveinsson S. Dempewolf H. Yang J. Y. Zhang D. Engels J. M. (2012). Ultra-barcoding in cacao (*Theobroma* spp.; *Malvaceae*) using whole cp genomes and nuclear ribosomal DNA. *Am. J. Bot.* 99 320–329. 10.3732/ajb.1100570 22301895

[B27] Katoh K. Kuma K. Toh H. Miyata T. (2005). MAFFT version 5: improvement in accuracy of multiple sequence alignment. *Nucl Acids Res.* 33 511–518. 10.1093/nar/gki198 15661851 PMC548345

[B28] Kempen H. M. Graf J. (1981). Weed seed production. *Proc. Western Soc. Weed Sci.* 34 78–81.

[B29] Kumar S. Steche R. G. Tamuram K. (2016). MEGA7: molecular evolutionary genetics analysis version 7.0 for bigger datasets. *Mol. Biol. Evol.* 33 1870–1874. 10.1093/molbev/msw054 27004904 PMC8210823

[B30] Laureto P. J. Barkman T. J. (2011). Nuclear and chloroplast DNA suggest a complex single origin for the threatened allopolyploid *Solidago houghtonii* (Asteraceae) involving reticulate evolution and introgression. *Syst. Bot.* 36 209–226. 10.1600/036364411X553289

[B31] Liu C. Shi L. Zhu Y. Chen H. Zhang J. Lin X. (2012). CpGAVAS, an integrated web server for the annotation, visualization, analysis, and GenBank submission of completely sequenced chloroplast genome sequences. *BMC Genomics* 13:715. 10.1186/1471-2164-13-715 23256920 PMC3543216

[B32] Lohse M. Drechsel O. Bock R. (2007). OrganellarGenomeDRAW (OGDRAW): a tool for the easy generation of high-quality custom graphical maps of plastid and mitochondrial genomes. *Curr. Genet.* 52 267–274. 10.1007/s00294-007-0161-y 17957369

[B33] Lowe T. M. Eddy S. R. (1997). tRNAscan-SE: a program for improved detection of transfer RNA genes in genomic sequence. *Nucleic Acids Res.* 25 955–964. 10.1093/nar/25.5.0955 9023104 PMC146525

[B34] Luo H. Shi J. Arndt W. Tang J. Friedman R. (2008). Gene order phylogeny of the genus Prochlorococcus. *PLoS One* 3:e3837. 10.1371/journal.pone.0003837 19050756 PMC2585141

[B35] Luo H. Sun Z. Arndt W. Shi J. Friedman R. Tang J. (2009). Gene order phylogeny and the evolution of methanogens. *PLoS One* 4:e6069. 10.1371/journal.pone.0006069 19562076 PMC2699033

[B36] Luo R. Liu B. Xie Y. Li Z. Huang W. Yuan J. (2012). SOAPdenovo2: an empirically improved memory-efficient short-read de novo assembler. *Gigascience* 1:18. 10.1186/2047-217X-1-18 23587118 PMC3626529

[B37] Marochio C. A. Bevilaqua M. R. R. Takano H. K. Mangolim C. A. Oliveira Junior R. S. Machado M. F. P. S. (2017). Genetic admixture in species of Conyza (Asteraceae) as revealed by microsatellite markers. *Acta Sci. Agron.* 39 437–445. 10.4025/actasciagron.v39i4.32947

[B38] Michael P. W. (1977). “Some weedy species of Amaranthus (amaranths) and *Conyza/Erigeron* (fleabanes) naturalised in the Asian-Pacific region,” in *Proceedings of the 6th Asian-PacificWeed Scial Socity Conference*, (Jakarta: Asian-Pacific Weed Science Society), 87–95.

[B39] Moretti M. L. Hanson B. D. (2016). Reduced translocation is involved in resistance to glyphosate and paraquat in *Conyza bonariensis* and *Conyza canadensis* from California. *Weed Res.* 57 25–34. 10.1371/journal.pone.0180794 28700644 PMC5507266

[B40] Nesom G. L. (2008). Classification of subtribe Conyzinae (Asteraceae: *Astereae*). *Lundellia* 11 8–38.

[B41] Noyes R. D. (2000). Biogeographical and evolutionary insights on Erigeron and allies (Asteraceae) from ITS sequence data. *Plant Syst. Evol.* 220 93–114. 10.1007/BF00985373

[B42] Parks M. Cronn R. Liston A. (2009). Increasing phylogenetic resolution at low taxonomic levels using massively parallel sequencing of cp genomes. *BMC Biol.* 7:84. 10.1186/1741-7007-7-84 19954512 PMC2793254

[B43] Peng Y. H. Lai Z. Lane T. Nageswara-Rao M. Okada M. Jasieniuk M. (2014). De novo genome assembly of the economically important weed horseweed using integrated data from multiple sequencing platforms. *Plant Physiol.* 166 1241–1254. 10.1104/pp.114.247668 25209985 PMC4226366

[B44] Salih R. H. M. Majesky L. Schwarzacher T. Gornall R. Heslop-Harrison P. (2017). Complete chloroplast genomes from apomictic *Taraxacum* (Asteraceae): identity and variation between three microspecies. *PLoS One* 12:e0168008. 10.1371/journal.pone.0168008 28182646 PMC5300115

[B45] Song H. Buhay J. Whitting M. Crandall K. (2008). Many species in one: DNA barcoding overestimates the number of species when nuclear mitochondrial pseuodgenes are coamplified. *Proc. Natl. Acad. Sci. U.S. A.* 105 13486–13491. 10.1073/pnas.0803076105 18757756 PMC2527351

[B46] Suzuki J. Y. Matsumoto T. K. Keith L. M. Myers R. Y. (2014). The Cp psbK-psbI intergenic region, a potential genetic marker for broad sectional relationships in *Anthurium*. *Hortscience* 49 1244–1252.

[B47] Thebaud C. Abbott R. (1995). Characterization of invasive *Conyza* species (Asteraceae) in Europe, quantitative trait and isozyme analysis. *Am. J. Bot.* 82 360–368. 10.1002/j.1537-2197.1995.tb12640.x

[B48] Wang X. Lu P. Luo Z. (2013). GMATo: a novel tool for the identification and analysis of microsatellites in large genomes. *Bioinformation* 9 541–544. 10.6026/97320630009541 23861572 PMC3705631

[B49] Weaver S. E. (2001). The biology of Canadian weeds. 115. *Conyza canadensis. Can. J. Plant Sci.* 81 867–875. 10.4141/P00-196

[B50] Wu H. (2009). “Biology of flaxleaf fleabane (Conyza bonariensis L. Cronq.),” in *The Biology of Australian Weeds* Vol. 3 ed. Panetta F. D. (Melbourne, VIC: R.G. and F.J. Richardson), 85–101.

[B51] Wu H. Walker S. Robinson G. (2010). Control of flaxleaf fleabane (*Conyza bonariensis* L. Cronq.) in wheat and sorghum. *Weed Technol.* 24 102–107. 10.1614/WT-09-043.1

[B52] Wu H. Walker S. Rollin M. J. Tan D. K. Y. Robinson G. Werth J. (2007). Germination, persistence, and emergence of flaxleaf fleabane (*Conyza bonariensis* [L.] *Cronquist)*. *Weed Biol. Manag.* 7 192–199. 10.1111/j.1445-6664.2007.00256.x

