# Supplementary material for: Species Identification of Conyza bonariensis Assisted by Chloroplast Genome Sequencing
Source: Front Genet. 2018 Sep 11;9:374. doi: 10.3389/fgene.2018.00374 (PMC6141629; doi:10.3389/fgene.2018.00374)
Supplement: Supplementary file 1 [file Table_1.DOCX]

**Supplemental Tables**

Table S1: *Conyza* species used for DNA barcoding analysis in present study

| **Species name** | **Sample ID** | **GenBank Accession No** | **Institution Storing the Specimens** | **Country** |
| --- | --- | --- | --- | --- |
| *Conyza sumatrensis* | ww14653 | MH559520 | Charles Sturt University Australia | Australia |
| *Conyza sumatrensis* | ww14666 | MH559521 | Charles Sturt University Australia | Australia |
| *Conyza bonariensis* | ww14668 | MH559522 | Charles Sturt University Australia | Australia |
| *Conyza bonariensis* | ww14669 | MH559523 | Charles Sturt University Australia | Australia |
| *Conyza canadensis* | ww14673 | MH559524 | Charles Sturt University Australia | Greece |
| *Conyza canadensis* | ww16837 | MH559525 | Charles Sturt University Australia | Australia |
| *Conyza bilbaoana* | ww16872 | MH559526 | Charles Sturt University Australia | Australia |
| *Conyza canadensis* | ww17609 | MH559527 | Charles Sturt University Australia | Australia |
| *Conyza canadensis* | ww17610 | MH559528 | Charles Sturt University Australia | Australia |
| *Conyza bonariensis* | ww17667 | MH559529 | Museo Botanico de Cordoba | Greece |
| *Conyza bonariensis* | ww17677 | MH559530 | Charles Sturt University Australia | Greece |
| *Conyza sumatrensis* | ww17678 | MH559531 | Charles Sturt University Australia | Greece |
| *Conyza sumatrensis* | ww17680 | MH559532 | Charles Sturt University Australia | Greece |
