# Supplementary material for: Species Identification of Conyza bonariensis Assisted by Chloroplast Genome Sequencing
Source: Front Genet. 2018 Sep 11;9:374. doi: 10.3389/fgene.2018.00374 (PMC6141629; doi:10.3389/fgene.2018.00374)
Supplement: Supplementary file 2 [file Table_2.DOCX]

**Supplemental Tables**

Table S2: Intergenic regions with size above 500 bp in *C. bonariensis* WW08606

| **Start** | **End** | **Length (bp)** |
| --- | --- | --- |
| trnN-GUU | *rps15* | 5,754 |
| *rps16* | *trnQ-UUG* | 1,895 |
| *trnT-GGU* | *psbD* | 1,354 |
| *psbE* | *petL* | 1,261 |
| *trnR-UCU* | *trnT-GGU* | 1,215 |
| *rpl16* | *rps3* | 1,190 |
| *atpI* | *atpH* | 1,127 |
| *rpl32* | *ndhF* | 1,079 |
| *trnE-UUC* | *rpoB* | 1,045 |
| *ycf15* | *rps12* | 1,015 |
| *accD* | *psaI* | 999 |
| *rps12* | *ycf15* | 956 |
| *ycf4* | *cemA* | 943 |
| *petB* | *petD* | 924 |
| *psbH* | *petB* | 921 |
| *trnL-UAG* | *rpl32* | 887 |
| *ycf3* | *trnS-GGA* | 871 |
| *clpP* | *clpP* | 819 |
| *psaA* | *ycf3* | 811 |
| *trnS-GCU* | *trnC-GCA* | 793 |
| *ycf3* | *ycf3* | 748 |
| *rpl20* | *rps12* | 735 |
| *atpB* | *rbcL* | 727 |
| *rpoC1* | *rpoC1* | 727 |
| *petA* | *psbJ* | 712 |
| *ndhB* | *ndhB* | 675 |
| *rpl2* | *rpl2* | 668 |
| *ycf15* | *trnV-GAC* | 654 |
| *trnF-GAA* | *ndhJ* | 652 |
| *atpF* | *atpF* | 634 |
| *clpP* | *psbB* | 624 |
| *trnV-GAC* | *ycf15* | 609 |
| *rps12* | *rps7* | 607 |
| *psbM* | *trnD-GUC* | 600 |
| *ndhB* | *trnL-CAA* | 594 |
| *rbcL* | *accD* | 582 |
| *trnL-CAA* | *ndhB* | 574 |
| *psbA* | *matK* | 557 |
| *trnC-GCA* | *petN* | 532 |
